# Over-Expression of 60s Ribosomal L23a Is Associated with Cellular Proliferation in SAG Resistant Clinical Isolates of *Leishmania donovani*

**DOI:** 10.1371/journal.pntd.0002527

**Published:** 2013-12-05

**Authors:** Sanchita Das, Priyanka Shah, Rajendra K. Baharia, Rati Tandon, Prashant Khare, Shyam Sundar, Amogh A. Sahasrabuddhe, M. I. Siddiqi, Anuradha Dube

**Affiliations:** 1 Division of Parasitology, CSIR-Central Drug Research Institute, Lucknow, India; 2 Molecular and Structural Biology, CSIR-Central Drug Research Institute, Lucknow, India; 3 Department of Medicine, Institute of Medical Sciences, Banaras Hindu University, Varanasi, India; Lancaster University, United Kingdom

## Abstract

**Background:**

Sodium antimony gluconate (SAG) unresponsiveness of *Leishmania donovani* (Ld) had effectively compromised the chemotherapeutic potential of SAG. 60s ribosomal L23a (60sRL23a), identified as one of the over-expressed protein in different resistant strains of *L.donovani* as observed with differential proteomics studies indicates towards its possible involvement in SAG resistance in *L.donovani*. In the present study 60sRL23a has been characterized for its probable association with SAG resistance mechanism.

**Methodology and principal findings:**

The expression profile of 60s ribosomal L23a (60sRL23a) was checked in different SAG resistant as well as sensitive strains of *L.donovani* clinical isolates by real-time PCR and western blotting and was found to be up-regulated in resistant strains. Ld60sRL23a was cloned, expressed in *E.coli* system and purified for raising antibody in swiss mice and was observed to have cytosolic localization in *L.donovani*. 60sRL23a was further over-expressed in sensitive strain of *L.donovani* to check its sensitivity profile against SAG (Sb V and III) and was found to be altered towards the resistant mode.

**Conclusion/Significance:**

This study reports for the first time that the over expression of 60sRL23a in SAG sensitive parasite decreases the sensitivity of the parasite towards SAG, miltefosine and paramomycin. Growth curve of the tranfectants further indicated the proliferative potential of 60sRL23a assisting the parasite survival and reaffirming the extra ribosomal role of 60sRL23a. The study thus indicates towards the role of the protein in lowering and redistributing the drug pressure by increased proliferation of parasites and warrants further longitudinal study to understand the underlying mechanism.

## Author Summary

Visceral Leishmaniasis (VL) is the most fatal form in Indian subcontinent. Till last few years, the treatment of the disease was done with Sodium antimony gluconate (SAG), the first line drug against VL. This, however, was severely eroded by the resistance developed by the parasite against it. In order to understand the underlying mechanism, earlier a proteomic analysis of SAG sensitive as well as SAG resistant isolates of *L.donovani* (Ld) was done in which 60s ribosomal L23a (Ld60sRL23a) protein, one of the essential member of translational machinery, was found to be over-expressed. To examine its role in SAG resistance mechanism, which is hitherto not known, 60sRL23a was characterized and over-expressed in the sensitive isolate of *L.donovani*. The sensitivity of these transfectants, was found to be decreased towards SAG. The growth curve of transfectants clearly showed its proliferation potential in both promastigote and amastigote forms. Cell cycle analysis of the transfectants further assured its rapid progression towards the G2/M phase. The above studies, thus, indicate that 60s RL23a regulates proliferation of *L.donovani* parasites and represents a unique strategy to resist SAG. 60sRL23a could be further explored as a potential drug target to strengthen the chemotherapy strategy against *L.donovani*.

## Introduction

Leishmaniasis is a neglected tropical disease, affecting almost, more than 10 million people around the world and ranks itself second to malaria in terms of mortality and morbidity. It is caused by an obligatory intracellular protozoan parasite of the genus *Leishmania* and has varied clinical spectrum from self healing skin ulcers to fatal visceral infection if left untreated. As vaccine against visceral form is still a distant proposition, treatment against Visceral Leishmaniasis (VL) solely relies on chemotherapy. Unfortunately, during the last decade Sodium Antimony Gluconate (SAG) which had a traditional background of sixty years of chemotherapy has been worn out due to the resistance developed against this drug. This has become a major obstacle to the treatment, especially in India, where more than 60% of VL patients are unresponsive to SAG treatment. Although, new drugs have become available in recent years for treatment of VL, they are also far from satisfactory [1]. This is due to increased relapse cases, lack of cost effectiveness and emerging resistance against them, as reported earlier [2]. Therefore, understanding the resistance mechanism could only strengthen the search for safe and wise chemotherapeutic strategies against VL.

SAG having Sb (V) is a pro-drug and requires biological reduction to active form i.e. Sb (III) in macrophage and/or amastigotes. Sb (III) has been reported to interact with several targets. Resistance in general has been understood as interplay among uptake, efflux and/or sequestration of active molecule and modulating gene expression levels [3]. Most of the drug resistance studies were done on the laboratory mutants as compared to the clinical isolates. Although some studies emphasized on field isolates but these were based on biochemical, biophysical and immunological analysis of resistant isolates but several questions remains unrequited regarding the parasite's modulation for SAG at molecular level [4], [5]. Actual scenario could be more lucid by exploring clinical isolates and characterizing the up regulated as well as down regulated proteins in the resistant strains. Several differential studies revealed many proteins under several metabolic pathways, proteins involved in maintaining redox balance, transporters and signaling pathways along with a large number of translational/ribosomal proteins indicating its possible role in resistance mechanism in the resistant clinical isolates [6], [7], [8]. Some of them are well characterized for their possible role in SAG resistance mechanism e.g. Trypanothione reductase, γ glutamyl cysteine synthtase, ornithine decarboxylase and ABC transporters but ribosomal proteins are yet to be explored for its involvement in drug resistance against VL [9]. 60s ribosomal L23a (60sRL23a) is one such over-expressed protein in SAG resistant strain of *Leishmania donovani* (Ld) identified through differential proteomics indicating its possible involvement in SAG resistance in *L.donovani* [10].

60sRL23a encoded protein is a component of 60s subunit of large subunit of ribosome. In eukaryotes, ribosome biogenesis is a coordinated assembly involving four ribosomal rRNA molecules and more than seventy ribosomal proteins. It was believed that ribosome were previously consists of RNA only and ribosomal protein appeared later in the evolution to facilitate the protein synthesis. However, ribosomal proteins have also been reported to regulate cell growth and apoptosis apart from their regular translational apparatus activity [11]. There are some circumstantial evidences available regarding ribosomal proteins acting as modulators and effectors of changes [12]. Ribosomal proteins may be meant for ribosome, but could be recruited for extra ribosomal functions [13]. In the present study for the first time involvement of over-expressed 60sRL23a in *in vitro* SAG resistance has been explored in clinical isolates of *L.donovani*.

## Materials and Methods

### Parasites

Clinical isolates used in this study were isolated from patients at the Kala –Azar Medical Research centre, Institute of Medical Sciences, Banaras Hindu University, Varanasi, India, and at its affiliated hospital at Muzaffarpur, Bihar, India. Clinical isolates were obtained prior to drug treatment from VL patients who had responded to chemotherapy by SAG and were designated as SAG-S (SAG-sensitive), whereas VL patients who did not respond to SAG were designated as SAG-R (SAG-resistant). Promastigotes of corresponding strains were harvested by transformation of amastigotes from the splenic aspirates of kala-azar patients. SAG-S isolates used in this study include 2001 (S1) whereas the three SAG-R isolates were 2039 (R1), 1216 (R2), 761 (R3). The isolates used in this study were anonymized. The Dd8 (S2) strain (MHOM/IN/80/DD8) was used as a reference strain in this study. The isolates were maintained in RPMI-1640 medium containing 10%FCS at 26°C (Sigma, USA) in 75 cm^2^ culture flask (Nunc). The virulence have been retained in parasites through regular passage through hamster, so as to maintain their chemosensitivity profiles that was measured periodically by amastigote macrophage J774A.1 infectivity assay as described elsewhere [14].

### SAG sensitivity of clinical isolates *in vitro* and *in vivo*

#### *In vitro* assay

Mouse macrophage cell line, J774A.1, was cultured in Dulbecco's modified Eagle's medium (DMEM)(Sigma, USA) in 16 well chamber slides (Nunc, USA) to a cell density of 10^5^ cells/well and infected with late log phase promastigotes (S1, S2, R1, R2, R3) at a multiplicity of infection of 10∶1 (parasite/macrophage) and incubated at 37°C in 5% CO_2_ for 8–12 h after which chamber slides were washed with PBS and finally the wells were supplemented with fresh medium. Different concentrations of SAG [Sb (V) (Albert David)] were added to the wells in triplicate and incubated for 48–96 h. Chamber slides were fixed in absolute methanol, stained with Geimsa and examined under oil immersion objective of light microscope. At least 100 macrophages were counted per well for calculating % infected macrophages. Percentage inhibition (PI) of parasite multiplication was calculated in comparison to untreated/control using the formula: PI = no. of parasite count from infected control – no. of parasites from the treated group/no of parasite count from infected control per 100 macrophages [15]. Sb (III) (Sigma) sensitivity of parasite isolates (Promastigotes) was analyzed as described elsewhere [16].

#### *In vivo* assay

Golden hamsters were infected intracadially with 1×10^7^ late log phase promastigote of clinical isolates (S1, S2, R1, R2, R3), per 0.1 ml of 1× PBS. Parasitic burden were assessed on day 20–25 post infection by performing splenic biopsies as described previously [17]. Once the infection sets in with promastigote form, further passages in hamsters were carried out with splenic amastigotes as described previously [18]. Animals carrying 20–30 days old infection were employed for SAG sensitivity assay. Infected hamsters (5 animals for each dose of different clinical isolates) were treated intraperitoneally (i.p.) with SAG at doses of 80, 40 and 20 mg/kg body weight. Five infected hamsters were kept as untreated control. Splenic biopsies were again performed on day 7 post treatment after administration of last dose of SAG. Parasitic burden of both treated and untreated were infected animals was assessed with smear touch blots of spleen of hamsters and percentage parasite inhibition in treated animals was calculated by as per formula described elsewhere [15].

### Cloning expression and purification of recombinant Ld60sRL23a

*L.donovani* genomic DNA was isolated from 10^8^ cultured promastigotes [19]. Genomic DNA was spooled and subjected to RNase (100 µg/ml) treatment. 60sRL23a gene was amplified using primers: forward 5′GGTACCATGCCTCCTGCTCAGAAG3′ and reverse5′ AAGCTTGACAAGACCGATCTT3′ and Taq DNA polymerase (Sigma Aldrich) lacking 3′-5′ exonuclease activity in a termocycler (Bio-Rad) under conditions at one cycle of 95°C for 5 min, 30 cycles of 95°C for 45 s, 54°C for 30 s, 72°C for 45 s, and finally one cycle of 72°C for 10 min. Amplified PCR product was electrophoresed in agarose gel and eluted from the gel by Gen Elute columns (Qiagen). Eluted product was cloned in pTZ57R/T (T/A) cloning vector (Fermentas) and transformed into competent DH5α cells. The transformants were screened for the presence of recombinant plasmids with 60sRL23a insert by gene specific PCR under similar conditions as previously mentioned. Isolated positive clones were sequenced from Chromous Biotech Pvt Ltd. (Bangalore) and submitted to the National Centre for Biotechnology Information http://www.ncbi.nlm.nih.gov/nuccore/GU121098.1 (accession no. GU121098.1). 60sRL23a was further sub cloned at the *Kpn*I and *Hind*III site of bacterial pTriEx4 (Novagen). The expression of 60sRL23a was checked in bacterial cell by transforming the 60sRL23a+pTriEx4 in *Escherichia coli* Rosetta Strain. The transformed cells were inoculated into 5 ml test tube culture medium (Luria Bertani) and allowed to grow at 37°C in a shaker at 220 rpm. Cultures in logarithmic phase (at OD_600_ of ∼0.5–0.6) were induced for 3 hrs with 1 mM isopropyl-ß-D-thiogalactopyranoside (IPTG) at 37°C. After induction cells were lysed in SDS- Sample buffer using 5× stock (0.313 M Tris-Hcl(pH 6.8), 50% glycerol, 10%SDS) [20]. Uninduced control culture was analyzed in parallel. These separated proteins from the polyacrylamide gel were transformed onto a nitrocellulose membrane in a semidry blot apparatus (Amersham) as described elsewhere [21]. Membrane was incubated for 1 h in blocking buffer followed by a 2 h incubation at room temperature with mouse anti-His antibody (Novagen) as primary antibody (1∶2500 dilution) and then incubated with goat anti-mouse HRP conjugate antibody (1/10,000: Bangalore Genie) for 1 h at room temperature. The blot was developed using an ECL kit (GE Biosciences). For purification of 60sRL23a 200 ml of LB medium containing 35 µg/mL of chloramphenicol and 35 µg/mL ampicillin were inoculated with *E.coli* Rosetta strain transformed with pTriEx4-Ld60sRL23a and grown to an O.D._600_ of, 0.6 and then induced by addition of 1 mM (IPTG, Sigma) then further incubated for an additional 4–5 h at 37°C. The rLd60sRL23a was purified by affinity chromatography using Ni^2+^ chelating resin to bind the His6- Tag fusion peptide derived from the pTriEx4 vector. The cell pellet was resuspended in 5 mL of lysis buffer (10 mM Tris-HCl (pH 8.0), 200 mM NaCl,) containing 1∶200 dilution of protease cocktail inhibitor (Sigma) and incubated for 30 mins on ice and the suspension was sonicated for 10×20 s (with 30 s interval between each pulse) on ice. The sonicated cells were centrifuged at 15,000 g for 30 min, and the supernatant was incubated at 4°C for 1 h with the 2 ml of Ni-NTA Superflow resin (Qiagen, Hilden, Germany) previously equilibrated with lysis buffer. After washing with buffer (10 mM Tris-HCl, 200 mM Nacl) containing different concentrations of imidazole i.e. 10, 20, 30 and 50 mM, the purified rLd60sRL23a was eluted with elution buffer (10 mM Tris-HCl, 200 mM NaCl, and 200 mM imidazole, pH 7.5). The eluted fractions were analysed by 12% SDS-PAGE and the gels were stained with Coomassie brilliant blue R-250 (Sigma-Aldrich, St.Louis, USA). The protein content of the fractions was estimated by the Bradford method using bovine serum albumin (BSA) as standard.

### Production of polyclonal antibodies against rLd60sRL23a and western blot analysis

The purified rLd60sRL23a was used for raising antibodies (Ab) in swiss mice. Swiss mice were first immunized using 25 µg of rLd60sRL23a in Freund's complete adjuvant. Twelve days after the first dose the mice were given 2 booster doses of 15 µg of the recombinant protein each in incomplete Freund's adjuvant at 15 days interval and blood was collected after the last immunization by sacrificing the mice for serum collection. For immunoblotting experiment, purified rLd60sRL23a protein and whole cell lysate (WCL) were resolved on 12% SDS-PAGE and transformed onto nitrocellulose membrane using a semi-dry blot apparatus (Amersham) [21]. After overnight blocking in 5% BSA, the membrane was incubated with antiserum to the rLd60sRL23a protein at a dilution of 1∶3000 for 120 min at room temperature (RT). The membrane was washed three times with PBS containing 0.5% Tween 20 (PBS-T) and then incubated with Rat anti-Mouse IgG HRP conjugate (Invitrogen, Carlsbad, USA) at a dilution of 1∶10,000 for 1 h at room temperature. Blot was developed by using diaminobenzidine+imidazole+H_2_O_2_ (Sigma).

#### Immunolocalization studies

The expression of 60sRL23a at the appropriate cellular localizations was confirmed by immunofluorescence using polyclonal Ab raised in swiss mice against r60sRL23a. Ld (S1) parasites were plated on four 18 mm cover slips (BLUE STAR) and fixed using 4% paraformaldehyde. Two of them were permeabilized by 0.5% Triton X-100 for 7 min followed washing with 1×PBS. One permeabilized and one non-permeabilized coverslips (positive controls) were incubated with the same primary antibody followed by washing with 1×PBS and then incubated with secondary anti-mouse FITC conjugate (Banglore Genei) for 1 h at room temperature. After washing in PBS, cover slips were mounted upside down on glass slides with Fluorescent Mounting Media (CALBIOCHEM) and visualized directly under fluorescence microscope (Eclipse 80i Nikon) using 100× oil objective (1.4 NA). Cells transfected with 60srL23a+pXG-'GFP+, were visualized directly under the same fluorescent microscope [15].

#### Homology modeling

Amino acid sequence of *L. donovani* 60sRL23a (Uniprot id: D1M863) was used for homology modeling. The Hidden Markov model based profile-profile alignment algorithm available at HHpred server on the bioinformatics toolkit platform of the Max Planck Institute for Developmental Biology (http://toolkit.tuebingen.mpg.de/hhpred) was used to find best template in PDB database for homology modeling with default settings [22]. HHpred uses HMM based profile-profile alignment method for identification and alignment of homologous protein. After ensuring high degree of accuracy in alignment, ten homology models were built using Modeller 9.10 with thorough optimization [23]. Final model was selected on the basis of DOPE score and structural validations were performed by using SAVS server. (http://nihserver.mbi.ucla.edu/SAVES_3/).

### Real time quantitative analysis of 60sRL23a expression in clinical isolates

10 million log phase parasites each of S1, S2, R1, R2, R3 were taken for RNA extraction. The freshly harvested promastigotes were immediately resuspended in Tri reagent (Sigma, Aldrich). RNA was isolated according to the manufacture protocol. Isolated RNA were treated with DNase and quantified in Gene-quant (Biorad). Total RNA (1 µg/reaction) was reverse transcribed using first-strand cDNA synthesis kit (Fermentas) and then cDNA was treated with RNase. For qRT-PCR primers were designed using Beacon Designer software (Biorad). qRT-PCR was carried out with 12.5 ml of SYBER green PCR master mix (TAKARA), 1 µg of cDNA, and primers at a final concentration of 200 nM in a final volume of 25 µl. PCR was conducted under the following conditions: initial denaturation at 95°C for 10 min followed by 40 cycles, each consisting of denaturation at 95°C for 1 min, annealing at 52°C for 1 min and extension at 70°C for 1 min followed by 80°C for 10 sec. 87 cycles of melt curve was set at 52°C for 10 sec. All quantification was normalized to the Ld-actin gene. A no-template control cDNA was included to eliminate contaminations or nonspecific reactions. The cycle threshold (CT) value was defined as the number of PCR cycles required for the fluorescence signal to exceed the detection threshold value (background noise). Difference in gene expression was calculated by comparative CT method [24]. This method compares test samples to the comparator sample and uses results obtained with a uniformly expressed control gene (Ld-actin) to correct for differences in the amounts of RNA present in the two samples being compared to generate a ΔCT value. Results are expressed as the degrees of difference between ΔCT values of test and comparator sample (S1) to get ΔΔCT i.e. [25]. Then the normalized expression ratio was calculated as 2^−ΔΔCT^ [26].

### Overexpression of Ld60sRL23a in sensitive strain of Ld

60sRL23a gene was amplified from 60sRL23a+pTriEx4 construct using primers: forward 5′ GGATCCATGCCTCCTGCTCAGAAGACC3′ and reverse 5′ GATATCGACAAGACGGATCTTGTTGGCAG3′ and then cloned into *Leishmania* expression vector pXG-'GFP+ at *BamHI* and *EcoRV* site [27]. Late log phase S1 promastigotes were washed with transfection buffer (21 mM HEPES,pH 7.5, 137 mM NaCl, 5 mM KCl, 0.7 mM Na_2_HPO_4_, 6 mM glucose). Transfection of the parasites with 20 µg of 60sRL23a+pXG-'GFP+ and pXG-'GFP+ alone, were carried out in 0.2 cm electroporation cuvet using a Gene Pulsar (Bio-Rad). Transfectants were allowed to recover for 24 h and then were selected for resistance to G418 at 5, 10, 20, 50 µg/mL.

### Growth profile of transfectants S1 (60sRL23a+pXG-'GFP+) and S1(pXG-'GFP+) -

2×10^6^ parasites (promastigote) were seeded. Counting of parasites (promastigote) for each transfectants was done for 8 days. Growth curve was plotted as number of parasites versus number of days. The proliferative potential of transfectants at amastigote stage was studied in macrophage amastigote (1∶8) system in chamber slide. Parasites in macrophages were counted by geimsa staining at 0 h, 6 h, 12 h, 24 h, 48 h.

#### Cell cycle analysis

Log phase promastigotes were synchronized with 0.3 mM hydroxyurea for 16 hrs. Then the parasites were washed to remove hydroxyurea. Cells were fixed at 0, 3, 6, 12 h in 70% ethanol for 2 h on ice and then resuspended in 0.5 mL PBS containing 0.5 mg PI and 50 mg RNase A and incubated for 1 h in dark at room temperature [28]. Data acquisition was carried out at FACS Calibur and analyzed using CELLQUEST software.

### Sb(III) and Sb(V) sensitivity profile of transfectants

Sb(III) and Sb(V) sensitivity profile of Transfectants growing at 0 µg, 20 µg, 50 µg were determined in the same procedure as it was done for clinical isolates.

### Sensitivity profile of transfectants towards other antileishmanial drugs

Transfectants (promastigotes) growing at 50 µg of G418 were assessed for their sensitivity towards miltefosine (SynphaBase), paramomycin (Sigma) and amphotericin B (Sigma) through flow-cytometry as described earlier [29].

#### Ethics statement

The clinical isolates were obtained from the splenic biopsies carried out on the Leishmania patients. The study was approved by the ethics committee of the Kala-azar Medical Research Centre, Muzaffarpur and CDRI. The patients provided a written consent and were informed before enrolment to this study. Experiments on the animals (hamsters) were performed following the approval of the protocol and the guidelines of Institutional Animal Ethics Committee (IAEC) of the CDRI which is adhered to National Guideline of CPCSEA (Committee for the Purpose of Control and Supervision on Experiments on Animals) under the Ministry of Environment and Forest, Government of India. The approval reference number 25/08/Para/IAEC dated 03.08.2011.

#### Statistical analysis

The 50% inhibitory concentrations (IC_50_s) of drugs were calculated by non linear regression obtained through log [inhibitor] vs. response- variable slope of log dose/response data on the drug [30]. The data were statistically analyzed by the one way ANOVA test and a post Tukey test and are presented as means and standard deviations (SDs) of three determinations from at least three independent experiments. AP value of <0.05 was considered significant.

#### Accession numbers

*Leishmania donovani* 60S ribosomal protein L23a gene, complete cds has been submitted to NCBI and has accession no GU121098.

## Results

### SAG sensitivity profile of clinical isolates in hamster and macrophages

**Figure 1:**
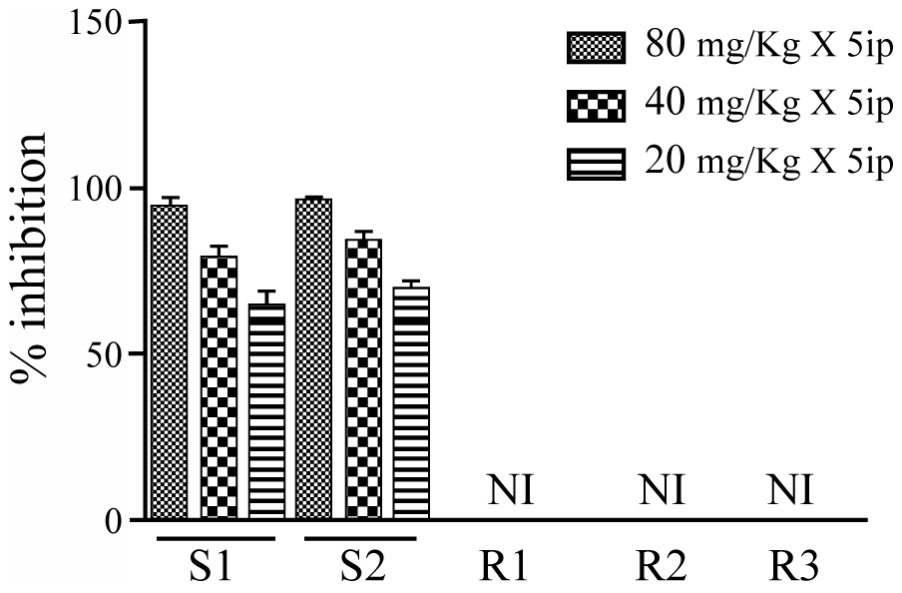
Efficacy of SAG (80, 40, 20 mg/kg) against *L.donovani* clinical isolates in golden hamsters: Parasitic burden was estimated by splenic biopsy on day +7 post treatment and percentage inhibition of parasite multiplication was calculated in comparison to parasitic burden of untreated animal. NI, no inhibition.

The *in vitro* and *in vivo* SAG sensitivity was assessed in clinical isolates isolated from SAG responsive and unresponsive patients from Muzaffarpur. The *in vitro* chemotherapeutic profile of clinical isolates was summarized in (Table 1). The *in vitro* SAG sensitivity assay was studied with both Sb (V) and Sb (III) and has been found to be correlated to each other as confirmed by the resistance index (Table 1). The chemotherapeutic sensitivity profiles of clinical isolates tested in hamster model showed the successful treatment of hamsters infected with S1 and S2 with standard dose of SAG (80 mg/kg×5 i.p.) with a percent inhibition (PI) of 94.97±2 for S1 and 96.915±0.84 for S2. SAG still exerted leishmanicidal action at 20 mg/kg×5ip with PI of 65.67±3.75 for S1 and 70.640±1.24 for S2. PI was 79.98±2.41 for S1 and 84.87±2.313 for S2 at 40 mg/kg×5 i.p. whereas SAG failed to inhibit the multiplication of R1, R2 and R3 even at higher doses (Figure 1).

**Table 1:** *In vitro* sensitivity profile of clinical isolates to Sb(V) and Sb(III).

			Mean Resistance index ± SD	
Strains	Collection Area	Clinical Drug Response	SbIII	SbV
S2	Laboratory Strain	Sensitive	1^a^	1^b^
S1	Muzaffarpur	Sensitive	0.767±0.150	0.919±0.099
R1	Muzaffarpur	Resistant	3.854±0.162	2.258±0.071
R2	Muzaffarpur	Resistant	3.617±0.064	2.653±0.113
R3	Muzaffarpur	Resistant	3.060±0.099	1.997±0.114

S, sensitive; R, resistant.

1^a^, IC_50_, 24.46±3.875 µg/mL.

1^b^, IC_50_, 94.12±2.099 µg/mL.

Sb(III) and Sb(V) resistance indices were calculated using the formula IC_50_ isolates/IC_50 Dd8_.

#### Molecular characterization of Ld60sRL23a

**Figure 2:**
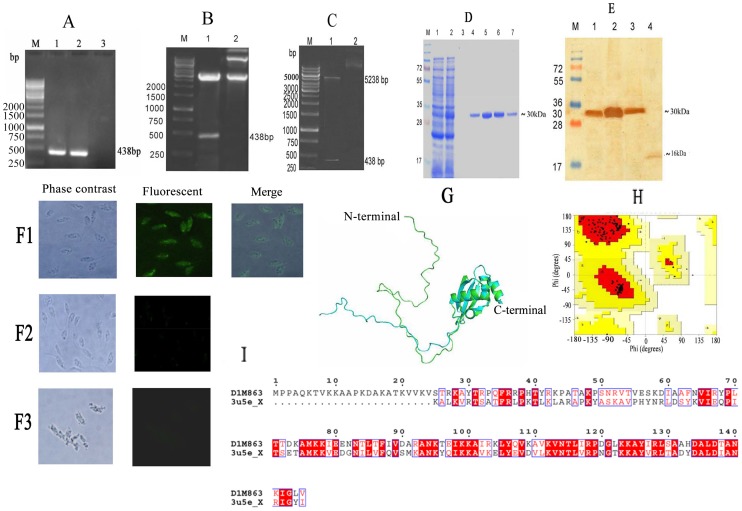
Molecular characterization of *L. donovani* 60s Ribosomal L23a (60sRL23a). (A) **PCR amplification of Ld60sRL23a**. M: 1 kb molecular mass marker; Lane 1&2: PCR amplificon of 60sRL23a; 3: negative control. (B) **Clone confirmation of Ld60sRL23a in TA vector**. M: 1 kb molecular mass marker; Lane1: *KpnI* and *HindIII* digested TA-Ld60sRL23a; Lane2: Undigested TA-Ld60sRL23a. (C) Clone confirmation of Ld60sRL23a in pTriEx-4 vector. M: 1 kb molecular mass marker; Lane1: *KpnI* and *HindIII* digested pTriEx-4 -Ld60sRL23a; Lane2: Undigested pTriEx-4-Ld60sRL23a. (D) **Expression (in** ***E.coli*** **), purification and elution of r60sRL23a** at 200 mM of imidazole concentration of 60sRL23a and separation in 12%SDS PAGE. M: Molecular wt. Markers, Lane 1 Whole cell lysate (WCL) of uninduced *E.coli* and Lane2: WCL of *E.coli* induced at 37°C and 1 mM IPTG; Lane3: wash fraction; Lane 4, 5,6and7: purified protein (E) **western blot analysis using anti r60sRL23a antibody**. M: Molecular mass marker; Lane1: WCL before IPTG induction; Lane2, WCL after IPTG (1 mM) induction at 37°C; Lane3: Purified protein; Lane 4: WCL of Ld. (F) **Immunolocalization of 60sRL23a in Ld**. F1: image of permeablized Ld with anti r60sRL23a and then FITC conjugated secondary antibody, F2: Image of non permeablized Ld with anti r60sRL23a and then FITC conjugated secondary antibody, F3: negative control. (G) and (H) **Superimposition of homology model** (in cyan color) on template (in green color) with RMSD of 0.667 Å. (I) **Alignment** between *L. donovani* 60S ribosomal protein L23a of Ld and 60S ribosomal protein L25 of *Saccharomyces cerevisiae*.

The 60sRL23a gene of *L.donovani* of 435 bp was successfully cloned in T/A vector (Figure 2A, 2B) and sequenced which has 97% identity to 60sRL23a of *L.major*, 95.86% to *L.mexicana* and 93.75% *L.infantum* (Table 2). It was further sub-cloned in bacterial expression vector pTriEx4 (Figure 2C), purified and eluted at 200 mM imidazole concentration. The size of the eluted r60sRL23a was ∼27 kDa (Figure 2D). Western blot analysis of *L.donovani* promastigote lysate was performed with the polyclonal anti-rLd60sRL23a antibody which detected protein in the whole cell lysate of Ld belonging to the molecular wt of 16 kDa. (Figure 2E). Immunolocalization studies further confirmed the cytosolic existence of the protein (Figure 2F).

**Table 2:** Percent identity of Ld60sRL23a with different sp. of *Leishmania* and *Homo sapiens*.

Species	Percent identity
*L.major*	97.86
*L.mexicana*	95.86
*L.infantum*	93.79
*L.braziliensis*	84.08
*H.sapiens*	46.94

Comparative modeling technique was attempted for obtaining three-dimensional coordinates of *L.donovani* 60S ribosomal protein L23a. 60S ribosomal protein L25 of *Saccharomyces cerevisiae* S288c (PDB id: 3U5E_chain X) was selected as template using hhpred server on the basis of lower E-value, P-value and higher score. This protein shows 43.80% similarity with *L.donovani* 60S ribosomal protein L23a sequence (Figure 2 I). The best molecular model showed the DOPE score of −10568.408203. Ramachandran plot obtained from Procheck shows high accuracy with 100% residues within the allowed region (figure 2G, 2H). Figure 2(I) shows the superimposed image of *L.donovani* 60sRL23a on *Saccharomyces cerevisiae* L25.

### Expression profile of 60sRL23a in different clinical isolates of *L.donovani*

**Figure 3:**
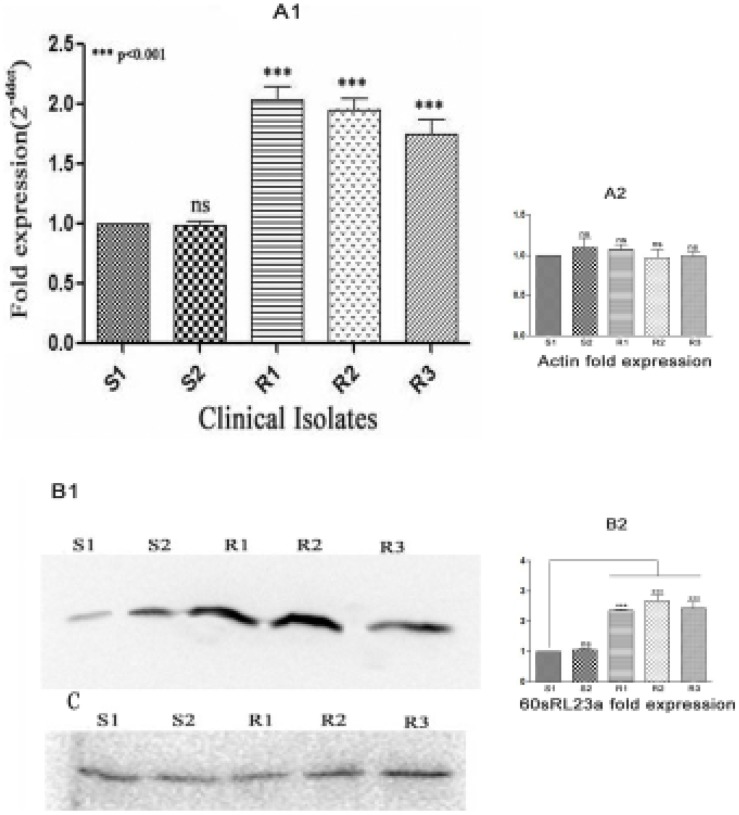
Differential expression of 60sRL23a in clinical isolates by real time PCR. (A1) **Relative expression of 60sRL23a** in different isolates to that of S1 as detected by real time PCR. The data are the mean of ± SD of three independent fold expression estimated through three independent RNA preparations. (Asterisks denotes highly significant differences from S1) (A2) **Relative fold expression of Ld-actin** (internal control) in several clinical isolates. (B1) **Differential pattern of expression of 60sRL23a** in different isolates as analyzed by Western blot. (B2) **Fold expression of 60sRL23a** in several clinical isolates as demonstrated through densitometric values (C) **Ld-actin** is used as internal control. (the experiment was repeated three times with three independent whole cell lysate preparations of promastigotes along with their respective loading control).

Since 60sRL23a was found to be over-expressed in the SAG resistant strains as identified through differential proteomics study it was further investigated for its expression profile in several resistant and sensitive strains of *L.donovani* through real-time PCR (Figure 3A). The study revealed the difference in the expression levels of 60sRL23a between the sensitive and resistant strains of *L.donovani*. There was ∼two fold increase in the expression of protein in resistant parasite (Figure 3B). Expression profile of 60sRL23a in protein level was further confirmed through western blot analysis with anti-r60sRL23a antibody and was found to replicate the response of real-time study as confirmed by the densitometric study through chemidoc software (BIORAD) using Ld-actin as internal control [31].

### Overexpression of 60sRL23a in *L.donovani* S1 parasites

**Figure 4:**
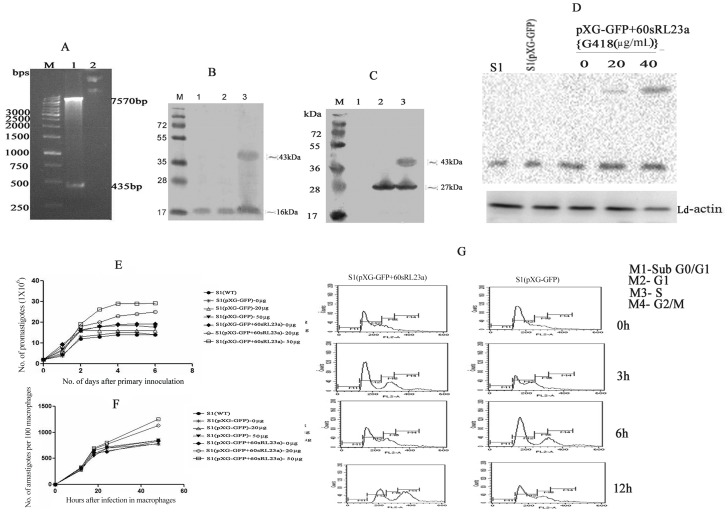
Over expression of 60sRL23a in S1. (A) **Clone confirmation of Ld60sRL23a** in pXG-'GFP+ vector. M: 1 kb molecular mass marker; Lane1: *BamH*I and *EcoRV* digested pXG-'GFP+Ld60sRL23a+; Lane2: Undigested pXG-'GFP+Ld60sRL23a+; (B) **Western blot analysis using anti r60sRL23a antibody**. M: Molecular mass marker; Lane1: WCL of S1(WT); Lane 2: WCL of S1(pXG-'GFP+); Lane 3: WCL of S1(pXG-'GFP+Ld60sRL23a+); (C) **Western blot analysis using anti GFP antibody**. M: Molecular mass marker; Lane1: WCL of S1(WT); Lane 2: WCL of S1(pXG-'GFP+); Lane 3: WCL of S1(pXG-'GFP+Ld60sRL23a); (D) **Western blot analysis of transfectants** (D1) at different concentration of G418 using anti r60sRL23a antibody (D2) at different concentration of G418 using anti Ld-actin antibody. (E) **Growth curve of transfectants** (Promastigote) (F) **Growth curve of transfectants** (amastigote) (G) **Cell cycle analysis of Transfectants**.

60sRL23a gene was further sub-cloned in *Leishmania* expression vector pXG-'GFP+ (Figure 4A) and was transfected in S1 to check whether this protein can alter the sensitivity profile of S1. The western blot analysis of the whole cell lysate of S1 [60sRL23a+pXG-'GFP+] parasite with anti-60sRL23a antibody revealed the identification of the protein at ∼43 kDa and ∼16 kDa mol wt. (Figure 4B), whereas with anti-GFP antibody the protein bands were detected at ∼43 kDa and ∼27 kDa (Figure 4C). The S1 lysate having vector alone [pXG-'GFP+] exhibited a band at mol wt of 27 kDa when analyzed with GFP antibody and at ∼16 kDa when analyzed with r60sRL23a antibody. This indicated towards the expression of GFP alone (Figure 4C). The increased expression pattern of the protein in S1 [60sRL23a+pXG-'GFP+] parasite has been observed with increased pressure of G418 at 0 µg/mL, 20 µg/mL and 50 µg/mL. (Figure 4D).

#### Growth profile of transfectants S1 [60sRL23a+pXG-'GFP+] and cell cycle analysis

Growth curve of transfectants revealed proliferation in S1 [60sRL23a+pXG-'GFP+] as compared to the transfectants having vector alone as well as WT (S1). 60sRL23a over expression was more when grown in presence of 50 µg/mL G418. The proliferation potential of S1 [60sRL23a+pXG-'GFP+] increases with the G418 concentration. The proliferative potential of transfectants was also replicated in amastigote stage (Figure 4F). The S1 transfected with empty vector has growth curve same as that of S1 (Figure 4E).

Since, the growth curve of both parasite stages exhibited higher proliferation, we investigated whether 60sRL23a protein is associated with accelerating cell cycle. Cell cycle analysis of transfectants revealed that S1 [60sRL23a+pXG-'GFP+] parasites showed an accumulation in ‘G2M’ phase as compared to S1 containing vector [pXG-'GFP+] alone when analyzed by flowcytometry at 0 h, 3 h, 6 h and 12 h. The S1(60sRL23a+pXG-'GFP+) has 9, 13, 18, 43% of parasites in ‘G2/M’ phase and 19, 20, 18, 23% of parasites in ‘S’ phase. Whereas S1(pXG-'GFP+) has 6, 10, 11, 13% of parasites in ‘G2/M’ phase and 14, 17, 23, 23% parasites in ‘S’ phase (Figure 4G). The proportion of S1(pXG-'GFP+) in G0/G1 phase was 1.14, 0.5, 0.31 and 0.02% whereas 1.19, 1.62, 1.42 and 1.10% of S1(60s+pXG-'GFP) in G0/G1 phase at different time interval.

### *In vitro* SAG sensitivity of transfectants

**Figure 5:**
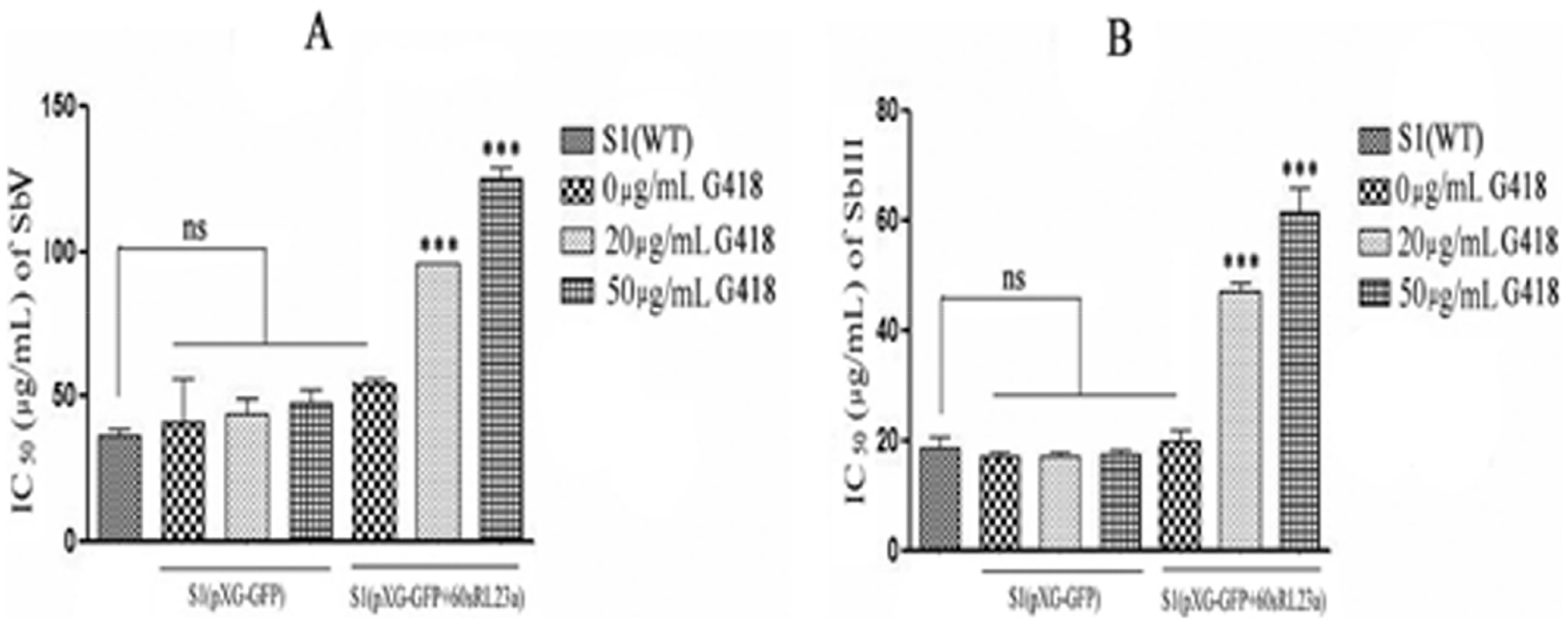
SAG sensitivity profile of transfectants. (A) with Sb(V); (B) with Sb(III) (The results are the mean ±SD of three independent IC_50_ estimation experiment of each group.)

*In vitro* SAG sensitivity of transfectants maintained at 0, 20, 50 µg of G418 were assessed with both Sb (V) in macrophage amastigote model and with Sb (III) in promastigotes. The sensitivity profile of the transfectants to Sb (V) and Sb (III) is depicted in (Figure 5A and 5B). S1 containing episomal over expressed 60sRL23a growing at 50 µg/mL of G418 has IC_50_ i.e. 158.066±4.28 for Sb(V) than of S1 expressing vector alone i.e. 92.506±5.7. S1 [60sRL23a+pXG-'GFP+] growing at 20 µg/mL of G418 also exhibited higher IC_50_ (123.2±5.117) as compared to S1 (78.55±6.5) containing vector alone. Whereas S1 [60sRL23a+pXG-'GFP+] growing at 0 µg/mL demonstrated the IC_50_ comparable to S1[pXG-'GFP+] and S1. The sensitivity of transfectants to Sb (III) and Sb (V) patterns were similar to each other. The Sb (III) IC_50_ of S1 [60sRL23a+pXG-'GFP+] i.e. 61.58±7.23 was found to be greater than S1 [pXG-'GFP+] i.e. 17.67±1.71 at 50 µg/mL of G418, whereas at 20 µg/mL of G418 it was 47.34±2.54 and S1 having empty vector has similar IC_50_ at all concentrations of G418. In absence of G418 the IC_50_ of transfectants were comparable.

### Sensitivity profile of transfectants to other antileishmanials (*in vitro*)

**Figure 6:**
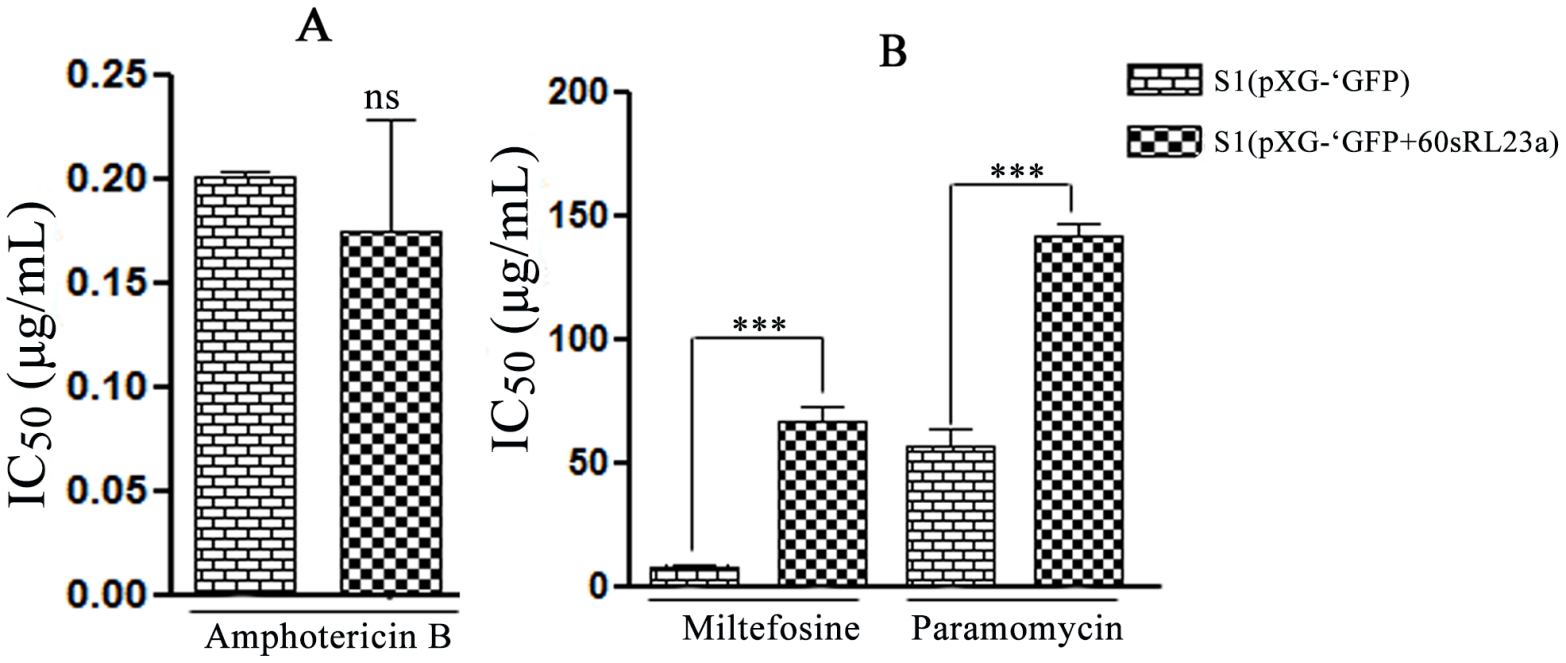
Sensitivity profile of transfectants with other antileishmanial drugs. (A) Amphotericin B, (B) Miltefosine and Paramomycin ( The results are the ±SD of three independent IC_50_ estimation experiment of each group.)

The transfectants were further checked for their sensitivity towards other antileishmanial compounds (miltefosine, paramomycin and amphotericin B) (Figure 6). S1 [60sRL23a+pXG-'GFP+] showed ∼7 fold decreased sensitivity towards miltefosine (67.38±9.64 µg/ml) and ∼2.4 fold towards paramomycin (142.16±8.76 µg/ml). S1 [pXG-'GFP+] demonstrated IC_50_ of 8.6±1.44 for miltefosine and 57.11±11.58 for paramomycin. Whereas the transfectant showed comparable IC_50_ for amphotericin B (0.201±0.003 µg/ml for S1 [pXG-'GFP+] and 0.1748±0.093 µg/ml for S1 [60sRL23a+pXG-'GFP+]).

## Discussion

The emergence of SAG resistance and the limited knowledge of the mechanism by which parasite acquire resistance are the major obstacle for the control of VL. Several SAG resistance studies were done on the basis of differential proteomics or/and transcriptomics and microarray to understand the parasite strategy to escape the drug pressure. These studies led to the identification of several proteins, playing crucial role in liberating the drug pressure, including ribosomal proteins [6]–[8]. . The enhanced expression of ribosomal proteins has been reported in tumors such as breast cancers, prostate cancers and hepatocellular cancers [32]. These ribosomal proteins have not been studied in detail in relation to SAG resistance in *Leishmania*. In this study, therefore, we have evaluated the involvement of 60sRL23a in SAG resistance using five clinical isolates which were isolated from kala-azar patients (Muzaffarpur) and their SAG sensitivity was further verified *in vitro* (macrophage and amastigote model) and *in vivo* (hamster model). SAG sensitivity profile of sensitive and resistant isolates *in vitro* and *in vivo* was comparable. S2 (Dd8), a WHO reference laboratory strain of *L.donovani* was used in this study to assess the resistance index of isolates. Resistance index of different resistant clinical isolates (R1, R2, R3) revealed that their response to Sb (V) and Sb (III) were similar. *In vivo* response of the resistant isolates to SAG in hamster model exhibited no inhibition even at higher concentration of SAG where as the S1, S2 exhibited 94.97±2.0% and 96.915±0.84% inhibition. The sensitive isolates responded even at the lower doses of SAG (20 and 40 mg/kg×5i.p.). *In vitro* and *in vivo* SAG sensitivity profile of all the isolates replicated the patients' response and confirmed the persistence of SAG response of clinical isolates even after several passages in hamster. In order to assess the association of 60sRL23a in SAG resistance we cloned, expressed and purified the protein which exhibited very close homology with *L.major* 60sRL23a to the tune of 95% and 45% identity with humans indicating towards the difference among the humans and parasite's entity. The protein's mismatched homology with humans can present the protein as a potential drug target. Immunoblot study of *L.donovani* promastigote lysate with the polyclonal anti-rLd60sRL23a antibody has revealed one dominant protein of 16 kDa mol wt. This protein was identified earlier at lower molecular weight range in proteomic studies which is approximately identical to its observed molecular mass [10]. The protein has been observed to have a cytosolic localization in the parasite, though earlier in the proteomic study it was identified in the membrane fraction. The protein is a well known member of large subunit of ribosomes so it is obvious for its attachment to endoplasmic reticulum thus it would have eventually been identified in the membrane fraction in the proteomic study. To study the expression level of 60sRL23a in different clinical isolates real time and immunoblot analysis was done and it revealed the 2 fold expression of the transcript and protein in the resistant strain as compared to the sensitive one, verifying the differential proteomics finding [10]. Differential regulation of ribosomal proteins has been utilized by cells to cater their needs such as replication, transcription, delaying apoptosis and proliferation, thus helping the cell to escape the stress conditions [33]. In this study whether 60sRL23a could modulate the SAG sensitivity profile of parasite, protein has been over expressed in S1 in pXG-'GFP+ vector having GFP tag in C-terminus end of the protein. Immunoblot analysis with 60sRL23a polyclonal antibody revealed the ∼43 kDa protein in the lysate of S1 transfected with 60sRL23a gene containing vector, verifying over expression of GFP tagged 60sRL23a. The immunoblot analysis with the GFP antibody identified two protein bands at ∼43 kDa and ∼27 kDa, indicating the detachment of GFP protein from combined entity of 60sRL23a+pXG-'GFP+ after expression (Figure 4C). This ensures the expression of protein returns in its original length after fusion protein expression. Transfectants expression profile in increasing G418 concentration indicated the increased protein expression pattern with increasing amount of G418 (Figure 4D) i.e. ∼ two fold expression of GFP tagged 60sRL23a in parasites growing in 50 µg/mL G418 as compared to parasites residing with 20 µg/mL G418, whereas no expression of GFP tagged 60sRL23a has been seen in 0 µg/mL of G418. Transfectants growth profile was analyzed by counting parasites per day for each transfectants and a clear proliferation has been observed in 60sRL23a over expressing parasites. The transfectants S1(60sRL23a+pXG-'GFP+) growing at 50 µg/mL of G418 has more proliferative potential as compared to transfectants growing at 0 µg/mL and 20 µg/mL of G418, indicating the increasing expression of protein leading the parasite proliferation. As the growth curve results exhibited greater number of parasites in transfectants over expressing 60sRL23a, thus the cell cycle progression of a synchronized transfected and control parasite population was analyzed to identify its relation with 60sRL23a expression. Parasites exhibited the tendency towards ‘G2/M’ phase as increasing number of parasites are seen in this phase wherein parasite progresses at different time intervals in compared to the control S1(pXG-'GFP+) one. The different proportion of both parasites [S1(pXG-'GFP+) and S1(60s+pXG-'GFP+)] in S phase were approximately same at different time interval. In G0/G1 phase number of parasites were decreased in S1 (60s+pXG-'GFP+) as compared to S1(pXG-'GFP+) revealing no arrest and a smooth progression to G2M phase. Whereas in G2M phase at 8 hrs and 12 hrs the S1(60s+pXG-'GFP+) progressively increased in comparison to S1(pXG-'GFP+) leading to rapid proliferation of parasites. Cell cycle progression pattern of transfectants reconfirmed the proliferation of S1(60sRL23a+pXG-'GFP+). Cellular proliferation as one of the extra ribosomal functions of ribosomal proteins have been reported to alter the cell cycle by interacting with cyclin-dependent kinases (Cdk) and regulatory molecules of cell cycle check points [34], [35]. Although Cdk are absent in *Leishmania* still apoptosis like cell death has been evidenced in *Leishmania* [36]–[38]. Transfectants were checked for their SAG sensitivity *in vitro* in macrophage-amastigote model Sb (V) as well as in the promastigotes for Sb (III) directly. Results revealed the *in vitro* SAG sensitivity of transfectants maintained at 0, 20, 50 µg of G418 were assessed with both SbV in macrophage amastigote model and with Sb (III) in promastigotes. The sensitivity profile of the transfectants to Sb (V) and Sb (III) is depicted in (Figure 5A, 5B). S1 containing episomally over-expressed 60sRL23a growing at 50 µg/mL showed 1.7 fold higher IC_50_ (158.066±4.28) for Sb (V) than IC_50_ (92.506±5.7) of S1 expressing vector alone. S1(pXG-'GFP+60sRL23a) growing at 20 µg/mL of G418 also exhibited higher IC_50_ (123.2±5.117) which is 1.5 fold as compared to S1(78.55±6.5). Whereas S1 (pXG-'GFP+60sRL23a) growing at 0 µg/mL of G418 demonstrated the IC_50_ comparable to S1 (pXG-'GFP+) and S1. The sensitivity of transfectants to Sb (III) and Sb (V) patterns were similar to each other. The Sb (III) IC_50_ (61.58±7.23) of S1 (pXG-'GFP+60sRL23a) was 3.5 fold higher than S1 (pXG-'GFP+) (17.67±1.71) at 50 µg/mL of G418, whereas at 20 µg/mL of G418 it was 2.7 fold higher. In absence of G418 the IC_50_ of transfectants were comparable. The IC_50_ of S1(pXG-'GFP+60sRL23a) growing at 50 µg/mL was 1.2 fold higher than the IC_50_ of transfectants growing at 20 µg/mL. This sensitivity profile of transfectants to Sb (V)/Sb (III)and increasing expression pattern of 60sRL23a in response to varying G418 concentration revealed the SAG sensitivity profile and 60sRL23a expression pattern is inversely correlated. The IC_50_ values of transfectants, were ∼1.3 to 1.8 fold higher for Sb (V) and ∼1.2 to 1.4 fold higher for Sb (III) to all the three resistant isolates, depicting the comparable resistance acquired by the transfectants to that of resistant isolates. Since SAG helps in sustaining innate as well as adaptive immunity against *Leishmania* by generating ROS and NO, higher proliferating capacity would increase the chances of the parasite to survive the intracellular host killing mechanism and combating the drug pressure [39]. Cellular proliferative potential of 60sRL23a and decreased SAG sensitivity of transfectants further emphasized the need to check its sensitivity profile for other antileishmanial drugs such as miltefosine, paramomycin and amphotericin B (Figure 6). Results revealed the decreased sensitivity of transfectants towards miltefosine and paramomycin. Whereas transfectants retained unaltered sensitivity towards amphotericin B. Paramomycin in general is known to inhibit protein synthesis by targeting ribosomal proteins and resistant strains of paramomycin revealed upregulated translational/ribosomal proteins to combat the drug pressure [8]. On the other hand the resistance mechanism of miltefosine involves several defect in inward translocation and increased efflux of drugs [40]. Since paramomycin is known to inhibit protein synthesis and the exact working mechanism of miltefosine and SAG is still unknown, the pathways of these drugs may do the cross talk somewhere or the toxicity of these drugs could have been overtaken by the parasite through increased cellular proliferation. In the light of above observation increased proliferative potential may strengthen the parasite to redistribute or lower the drug pressure hence providing a prospect to escape the drug pressure. Amphotericin B being the most successful drug among these and no resistance cases reported till date, further revealed its unique mechanism unbeatable by the parasite. Despite of its peerless therapeutic results, its toxicity and cost factors further compelled us to rejuvenate the safe traditional drugs. Down regulation of 60sRL23a could validate the finding of the present study but as RNAi machinery is absent in *L.donovani* and only knockout remains the only way to study the down regulation effect of the gene, but knockout 60sRL23a would be futile due to multicopy of the gene present in the genome of *Leishmania*. Presence of multicopy of 60sRL23a again indicates the protein to be an essential component of parasite that could be used by the parasite in different ways as and when so ever needed. Hence this study could only confirm the after effects of up regulation of 60sRL23a. Study further revealed that parasite could use its usual protein to perform an unusual function such as cellular proliferation to combat pressure of different drugs carrying out different anti-parasitic pathway. Indian subcontinent is now relying on several other drug combinations other than SAG, but parasites had developed resistance against these drug combinations under laboratory conditions [41]. To win the battle against Leishmaniasis searching new drugs or combinations against *Leishmania* is not sufficient but our understanding for the resistance mechanism has to be explored enough to strengthen the new chemotherapeutic strategy. SAG has not been in use for some time on the Indian subcontinent, and although the removal of drug pressure is expected to allow the return of the sensitive parasites by natural selection, although this is not universally accepted [42], [43], [4], [5]. Our understanding regarding resistance mechanism is in its infancy, this study will help to focus on the substantial role played by the ribosomal proteins in disease progression by assisting the parasite to escape the drug pressure.
